# Can the Skeletal Muscle Carnosine Response to Beta-Alanine Supplementation Be Optimized?

**DOI:** 10.3389/fnut.2019.00135

**Published:** 2019-08-27

**Authors:** Pedro Perim, Felipe Miguel Marticorena, Felipe Ribeiro, Gabriel Barreto, Nathan Gobbi, Chad Kerksick, Eimear Dolan, Bryan Saunders

**Affiliations:** ^1^Applied Physiology and Nutrition Research Group, Rheumatology Division, Faculdade de Medicina FMUSP, School of Physical Education and Sport, University of São Paulo, São Paulo, Brazil; ^2^Exercise and Performance Nutrition Laboratory, Lindenwood University, St. Charles, MO, United States; ^3^Institute of Orthopaedics and Traumatology, Faculty of Medicine FMUSP, University of São Paulo, São Paulo, Brazil

**Keywords:** optimizing supplementation, muscle carnosine content, metabolism, buffering, modifying factors

## Abstract

Carnosine is an abundant histidine-containing dipeptide in human skeletal muscle and formed by beta-alanine and L-histidine. It performs various physiological roles during exercise and has attracted strong interest in recent years with numerous investigations focused on increasing its intramuscular content to optimize its potential ergogenic benefits. Oral beta-alanine ingestion increases muscle carnosine content although large variation in response to supplementation exists and the amount of ingested beta-alanine converted into muscle carnosine appears to be low. Understanding of carnosine and beta-alanine metabolism and the factors that influence muscle carnosine synthesis with supplementation may provide insight into how beta-alanine supplementation may be optimized. Herein we discuss modifiable factors that may further enhance the increase of muscle carnosine in response to beta-alanine supplementation including, (i) dose; (ii) duration; (iii) beta-alanine formulation; (iv) dietary influences; (v) exercise; and (vi) co-supplementation with other substances. The aim of this narrative review is to outline the processes involved in muscle carnosine metabolism, discuss theoretical and mechanistic modifiable factors which may optimize the muscle carnosine response to beta-alanine supplementation and to make recommendations to guide future research.

## Introduction

Carnosine is a histidine-containing dipeptide formed by beta-alanine (BA) and L-histidine that is abundant in human skeletal muscle (1). It performs a number of roles which may impact exercise such as antioxidant activity (2–5), antiglycation effects (6), enhanced calcium sensitivity (7, 8), and hydrogen ion (H^+^) buffering (9–11). In particular, the biological function of carnosine as a muscle buffer makes it an interesting compound for high-intensity exercise since performance during this type of activity may be influenced by H^+^ accumulation and can be improved by increasing buffering capacity (12). Accordingly, carnosine continues to attract interest due to its potential ergogenic benefits, with numerous investigations specifically focused on increasing its intramuscular content to optimize performance (13).

Beta-alanine is a non-proteogenic amino acid and the limiting factor for carnosine formation in the skeletal muscle (1). Chronic supplementation of BA between 4 and 24 weeks appears to be safe (14, 15) and can increase skeletal muscle carnosine content by up to 200% (16). Strong evidence supports the ergogenic role of BA supplementation for high-intensity exercise with meta-analytical data demonstrating its efficacy, particular during exercise 30 s to 10 min in duration (13). Despite growing evidence supporting the use of BA to enhance exercise performance, the individual response of muscle carnosine to supplementation is highly variable (16) and the amount of ingested BA converted into muscle carnosine appears to be low (17–19). Little is known about modifiable factors that may potentially influence the response of muscle carnosine content to BA supplementation. These factors include dose, duration, meal co-ingestion, co-supplementation with other compounds, and exercise. Enhanced understanding of these factors is of interest to athletes, support staff and researchers, as greater increases in muscle carnosine are associated with greater improvements in exercise capacity (16, 20). The aim of this narrative review is to outline the processes involved in muscle carnosine metabolism, discuss theoretical and mechanistic modifiable factors which may optimize the muscle carnosine response to BA supplementation, and to make recommendations to guide future research in this area.

## Muscle Carnosine Metabolism

Carnosine homeostasis is dependent on its synthesis from, and degradation to, its constituent amino acids. Carnosine is synthesized from BA and L-histidine in a reaction catalyzed by the non-specific enzyme carnosine synthase (*CARNS*), an enzyme located in skeletal muscle (21). Importantly, beta-alanine has a high affinity (K_m_, 1.0–2.3 mM) for carnosine synthase (22) along with a low muscle content (~0.2 mmol·kg^−1^ww) (23); histidine, on the other hand, is found in high concentration in muscle (~0.4 mmol·kg^−1^ww) (24) but has a low K_m_ (16.8 μM) for carnosine synthase (25). These data indicate that BA is the rate-limiting amino acid to muscle carnosine synthesis, a finding that is corroborated by supplementation studies that show that BA alone is similarly effective at increasing muscle carnosine content, than an equivalent dose of BA delivered in carnosine (which comprises both BA and histidine) (1).

**Figure 1:**
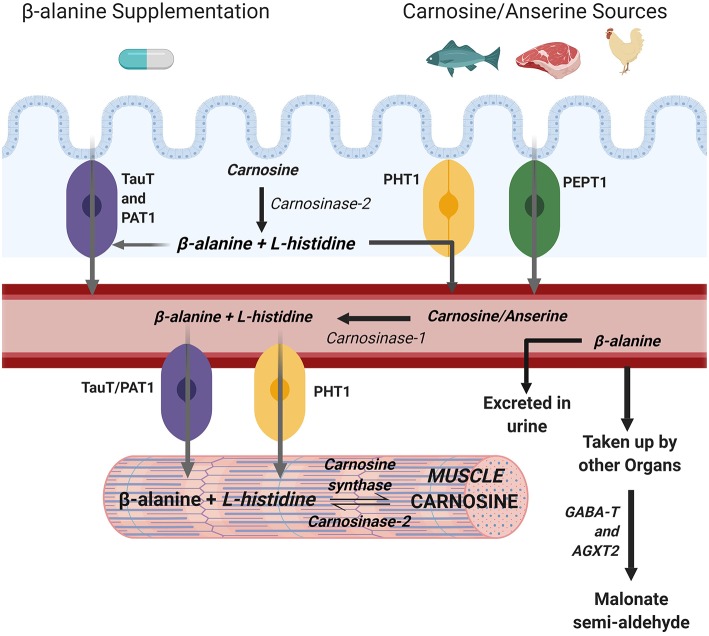
Metabolism of muscle carnosine. Created with BioRender.

Carnosinase is a hydrolytic enzyme found in serum and tissue (26) that actively degrades carnosine into its constituent amino acids (27). Serum carnosinase (also known as carnosinase-1) is highly specific for carnosine while carnosinase found in tissue (also known as carnosine-2) has a broader substrate specificity (28). Despite its presence in skeletal muscle as a cytosolic non-specific dipeptidase, carnosinase-2 functions optimally at pH 9.5 (26, 29) which is far in excess of the pH 7.4 typically encountered in human muscle meaning it has little influence in muscle. The presence of carnosinase in the gastrointestinal tract (30) means that some ingested carnosine, or histidine containing dipeptide analogs such as anserine or balenine (28), may be hydrolysed to BA and histidine before reaching the blood stream. Nonetheless, most carnosine will reach the blood stream where carnosinase-1 is highly present and active in humans, meaning that the carnosine that reaches the bloodstream is immediately hydrolysed into BA and histidine. Indeed, very little carnosine is found in human blood (31) and carnosinase activity is considered the main determinant of circulating carnosine levels following dietary carnosine ingestion (32). The constituent amino acids can then be transported to the muscle (Figure 1).

The uptake of BA into muscle is primarily mediated by *TauT*, a specific β-amino acid transport protein also responsible for the uptake of taurine into muscle that is dependent upon stoichiometric concentrations of both Na^+^ and Cl^−^ in a 2:1:1 (Na^+^:Cl^−^:β-amino acid) ratio (33). The BA transporter *TauT* into muscle cells has a K_m_ of 40 μM (34) which is relatively high compared to the <0.5 μM of BA typically found in blood (1) meaning that circulating levels must be increased to augment transport into muscle. Another transporter, *PAT1*, also transports BA into muscle although its contribution appears minimal compared to *TauT* (35). For these reasons, it is commonly accepted that the transport of BA into muscle is predominantly determined by the *TauT* transporter. While several non-specific peptide transporters (*PEPT1, PEPT2, PHT1, PHT2*) exist which can transport carnosine and its methylated analogs, only *PHT1* is found in abundance in human skeletal muscle and PEPT2 to a lesser extent (35).

Endogenous production of BA is low and occurs primarily inside the liver through the degradation of uracil (36). For this reason, dietary sources of histidine containing dipeptides (e.g., carnosine, anserine, balenine) such as meat, fish, and poultry [e.g., 200 g chicken breast contains ~800 mg of BA (1)] may be a determinant of muscle carnosine content (37). In support of this, vegetarians, whose only source of BA is endogenous production, have significantly lower muscle carnosine content compared to their omnivorous counterparts (38). However, omnivores who were put on a 6-month vegetarian diet did not reduce their muscle carnosine stores, suggesting carnosine homeostasis is tightly regulated and not entirely dependent on dietary intake (39). Nonetheless, it is unquestionable that BA intakes in excess of dietary intake are required to elicit significant carnosine increases (39), meaning supplementation with BA is the most effective and practical means by which to increase muscle carnosine content.

## The Influence of Beta-Alanine Supplementation on Muscle Carnosine Metabolism

The first study to show that BA could increase the intramuscular carnosine pool measured a +40–60% increase in carnosine content of the *m. vastus lateralis* (1), as measured by high-performance liquid chromatography (HPLC) of muscle biopsy samples. Numerous studies have corroborated these findings using HPLC (16, 20, 23, 24, 40) and proton magnetic resonance spectroscopy (^1^H-MRS) (41–46). Almost all individuals across these independent studies showed increases in muscle carnosine following a period of BA supplementation although there is a large variability in the magnitude of this response, both between and within studies. Data from our laboratory has shown maximal absolute increases of between +17 to +41 mmol·kg^−1^dm (+59 to +200%), highlighting the range of change between individuals. Variable responses are likely due to a combination of modifiable (i.e., dose, duration, co-supplementation, etc.) and non-modifiable (i.e., age, gender, disease) factors, although herein we will focus on the modifiable factors through which individuals might optimize muscle carnosine loading. Surprisingly, despite consistent and large increases in different muscle groups with BA supplementation, evidence suggests actual incorporation of BA into muscle carnosine is low. The amount of BA ingested that is converted into muscle carnosine is only about 3–6% (17–19), meaning that upwards of 90% of ingested BA is directed toward other physiological outcomes, which may include transamination and oxidation (47), while small amounts (~3%) are also lost via the urine (1). Understanding of the primary mechanisms by which increased BA availability increases muscle carnosine content is an essential step to see if its incorporation into muscle can optimized, while determination of the importance of these alternative pathways through which the majority of BA is metabolized may provide further scope for investigation.

It appears reasonable to expect that any changes in muscle carnosine content would be paralleled by changes in the proteins involved in its metabolism. Everaert et al. (35) showed upregulation of several genes encoding proteins and enzymes involved in carnosine homeostasis in response to 8 weeks of BA supplementation in mice. Specifically, gene expression for the enzymes relating to BA transport into muscle (*TauT*), synthesis of muscle carnosine (*CARNS*), and the deamination of BA (*ABAT*) increased expression, suggesting an important role for these proteins in increasing muscle carnosine content. The only study to measure changes in gene expression with BA supplementation in humans showed a chronic downregulation of *TauT* during 24-weeks of supplementation at 6.4 g·day^−1^, but no change in any other genes (16). It currently remains unclear why these two studies showed such contrasting results in gene expression following BA supplementation, particularly in reference to *TauT*. A key difference may be the timing of muscle sampling as it is unclear when dissection of the mice was performed relative to the last BA dose (35) while the human samples were always taken at least 4 h after the last ingested dose of BA. The time course response of carnosine-related gene expression following acute BA supplementation should be determined to further understand these findings since a single end-point biopsy following an intervention can influence the inferences made (48).

Muscle carnosine loading is most pronounced during the initial weeks of supplementation, after which increases in muscle carnosine content appear to slow. This is certainly true of the first vs. subsequent 12 days of supplementation (40), and the first 4 weeks compared to the remaining 20 weeks of supplementation (16). This slowing may be due to a decreased transport of BA into muscle, suggested by the downregulation of *TauT* gene expression (16). Despite this and as reported previously, intramuscular carnosine levels follow a progressive increase as long as supplementation continues whereby reported intramuscular carnosine content was greater after 20 and 24 weeks of supplementation when compared to 8 weeks of supplementation (16). In fact, several individuals showed substantial increases (> +6 mmol·kg^−1^dm) in the final 4 weeks of supplementation and it is likely that further increases in carnosine would have occurred if supplementation had continued. It seems possible, therefore, that *TauT* downregulation may attenuate the rate of carnosine synthesis in response to continued supplementation, but it does not block it completely. Further work should determine the true contribution of *TauT* to muscle carnosine increases with BA supplementation.

Beta-alanine can be transaminated into malonate semi-aldehyde by the enzymes GABA-T and AGXT2 for further metabolism within the citric acid cycle; these enzymes are highly expressed in kidney and liver of mice, but exhibit low expression in muscle (47). Low dietary intake of BA (0.1% BA in drinking water) in mice did not increase circulating BA or muscle histidine-containing dipeptide content (47), although when this low BA dose was provided alongside simultaneous inhibition of these BA transaminating enzymes, this led to increased circulating BA, and histidine-containing dipeptide content was increased in muscle and heart. These data suggest that low doses of BA may be entirely transaminated by highly active transaminating enzymes, leading to minimal to no changes in circulating BA or muscle histidine-containing dipeptide content, but should saturation of these enzymes occur, significant increases in the tissue concentrations of histidine-containing dipeptides can occur. The authors suggest that saturation of these enzymes is unlikely to occur with normal human dietary patterns, perhaps explaining the relative stability of muscle carnosine over time (42). It is possible however, that acute dietary ingestion via meat or fish may be sufficient to saturate these enzymes, since omnivores have higher muscle carnosine content than vegetarians (38). Certainly, it appears more than likely that under conditions of excess BA availability, such as supplementation, enzyme saturation occurs leading to increased circulating levels of BA and eventual uptake into skeletal muscle resulting in elevated intramuscular carnosine content. Since doses of BA as low as 1.6 g·day^−1^ lead to increases in muscle carnosine content (46), the likelihood that BA supplementation at these doses do indeed saturate these transaminating enzymes is high. The relevance of these alternate pathways of BA transamination may be an avenue of interest for further investigation.

It is currently unknown to what extent the acute plasma BA response to supplementation is related to chronic changes of carnosine in muscle when BA is ingested over an extended period. It could be hypothesized that greater increases in circulating BA may be due to lower transamination and, thus, may result in larger increases in muscle carnosine content. Supporting this, the carnosine and anserine concentration of murine skeletal and heart muscle appears dependent upon the circulating availability of BA (47). In humans, Stautemas et al. (49) showed a large inter-individual variability in the pharmacokinetic plasma BA profile following an acute absolute 1,400 mg dose of BA. Importantly, the high variability in plasma BA was not reduced after a dose relative to body mass. It is known that the time course plasma profile following an acute dose of BA appears stable throughout a period of chronic supplementation (1). Unfortunately, neither of these studies related chronic changes in muscle carnosine to the acute plasma BA profile, which may provide answers as to the importance of this initial acute plasma response to predict chronic changes and may direct future research in the area.

## Modifiable Factors Influencing the Increases in Muscle Carnosine Content With Beta-Alanine Supplementation

### Dose and Duration

The largest contributing factors to changes in muscle carnosine content appear to be the daily dose provided and the duration of supplementation. Doubling of the BA dose (12 vs. 6 g·day^−1^) halves the time taken to reach the same increases in the *m. vastus lateralis* (50). Similarly, Stellingwerff et al. (46) showed two-fold greater increases in carnosine of the *tibilias anterior* and *gastrocnemius* at a higher dose of 3.2 compared to 1.6 g·day^−1^ of BA for 4 weeks. Moreover, when supplementation was continued at 1.6 g·day^−1^ in both groups up to 8 weeks, muscle carnosine also continued to increase. Thus, there is strong evidence to show that a higher dose and/or longer supplementation period leads to greater accumulation of muscle carnosine. The muscle carnosine response to supplementation was initially proposed to be linearly related to the total amount of BA consumed (51). However, although doubling the dose appears to double the increases in muscle carnosine content during the first 2–4 weeks of supplementation (46, 50), a higher dose taken for a longer period (6.4 g·day^−1^ for 24 weeks) shows a slowing over time (16), suggesting this response is not linear.

Spelnikov and Harris (52) proposed a mathematical model describing the kinetics of carnosine accumulation in human skeletal muscle based on its rate of synthesis and decay. Using existing data, the model estimates that the rate of synthesis of carnosine in human skeletal muscle is constant over time for any given dose of BA, but that the rate of decay will change according to first-order kinetics (52). The washout of muscle carnosine has been shown to occur over several weeks to months before returning to similar pre-supplementation levels when supplementation ceases (42, 46), and could occur due to a number of reasons including transmembrane leakage and the formation of adducts with carbonyl groups, reactive oxygen species and reactive nitrogen species (5, 28). Based upon these parameters, the time course model of muscle carnosine changes predicts that with any BA dose, saturation for that specific dose will occur over time with continual supplementation. It must be noted that this model is currently speculative and the dose that will cause absolute saturation of carnosine in muscle is unknown; there are no known reports of muscle carnosine saturation in humans. Although the model predicts that a certain level of saturation will occur according to the continuation of supplementation at any specific dose, the first weeks of supplementation appear most susceptible to increases in muscle carnosine content (16) and thus the period most likely to be amenable to optimisation in BA supplementation.

### Beta-Alanine Formulation

Current recommendations for beta-alanine ingestion is for it to be taken in staggered doses of 800–1,600 mg every 3–4 h over the day in order to reduce the incidence and severity of paraesthesia, an uncomfortable tingling sensation on the skin that can last up to an hour (1). Although the exact cause of paresthesia is unknown, it is thought to be due to BA activated strychnine-sensitive glycine receptor sites, associated with glutamate sensitive N-methyl-D-aspartate receptors in the brain and central nervous system (53) and the mas-related gene family of G protein coupled receptors, which are triggered by interactions with BA (54). Given that the development of paresthesia is closely related to the time-to-peak beta-alanine concentration in blood following ingestion (1), sustained-release BA formulations have been developed to avoid this side-effect. Such sustained-release formulations directly reduce the symptoms of paresthesia and allow greater single tolerable doses of BA, which in turn will allow larger daily doses. This can lead to greater increases in muscle carnosine in the initial period of supplementation due to higher daily doses (24, 50). In support of this, symptoms of paresthesia while ingesting individual doses of 4 g of BA in sustained-release form were not different from those experienced with 2 g doses (50), meaning greater daily doses could be ingested without further discomfort leading to larger gains in muscle carnosine.

A study by Decombaz et al. (55) showed that ingestion of 1.6 g of BA in slow-release tablets resulted in slower absorption kinetics and improved whole body retention of BA, as measured by urinary excretion of BA, compared to the same dose in aqueous solution. Greater retention of BA suggests that supplementation in a sustained-release format might lead to greater increases in muscle carnosine compared to an instant release (e.g., powder) formulation, although the authors did not measure muscle carnosine in this study. Stegen et al. (19) did not show any differences in muscle carnosine increases in the *m. gastrocnemius* and *m. soleus* between individuals supplementing with 4.8 g·day^−1^ powder or sustained-released BA for 5 weeks. Varanoske et al. (24) compared sustained-release and rapid-release formulations of BA, providing volunteers with 6 g·day^−1^ for 28 days. Muscle carnosine content was significantly increased in the group consuming the sustained-release formulation while, perhaps surprisingly, no significant changes were shown with rapid-release supplementation despite a ~38% increase. However, the ~16% difference in elevation of muscle carnosine between the two formulations did not reach statistical significance, perhaps due to the small sample size or the short supplementation period. In fact, forward projecting the increases in muscle carnosine using a mathematical model (52) suggested that large differences would be found between formulations within 100 days of supplementation. However, these data are highly speculative and can only be proven with further research. As it stands, there is some evidence to suggest that supplementation with slow-release BA may enhance muscle carnosine increases relative to an instant-release formulation although this is more likely due to an increased tolerance allowing greater daily doses without the incidence of uncomfortable side-effects. More long-term studies are warranted to evaluate whether the same daily dose in different formulations leads to distinct increases in muscle carnosine content.

### Dietary Influences

The timing of ingestion is considered an important factor which may affect the efficacy of many dietary supplements (56, 57). Since BA is ingested at several timepoints throughout the day, it could be important to determine whether the timing of supplementation may influence the subsequent increased in muscle carnosine, particularly around meals and training. It has been suggested that co-ingestion of BA with carbohydrates or a carbohydrate-rich meal may lead to greater muscle carnosine increases than ingesting BA between meals (19) because the carbohydrate-mediated release of insulin upregulates the activity and content of the sarcolemmal Na^+^/K^+^-ATPase pumps (58, 59). Since the BA transporter *TauT* is dependent on sodium and chloride co-transport (34), muscle BA uptake and subsequently carnosine synthesis may be increased due to the action of insulin. In support of this theory, it is well-established that creatine uptake into muscle, which is also sodium-dependent, can be heightened when supplementation occurs alongside the intake of high glycaemic index carbohydrates (60, 61).

To date, only one study has investigated the potential influence of insulin on muscle carnosine increases with BA supplementation using a two-part investigation (19). Firstly, acute determination of whole-body BA retention showed no difference when BA was ingested in a fasted state or when co-ingested with two energy-rich carbohydrate bars. In Part B, participants ingested BA at 3.2 g·day^−1^ for 6–7 weeks, separated into two groups who were required to ingest the supplement with (co-ingestion) meals or between meals. Meal co-ingestion enhanced muscle carnosine loading in the soleus muscle, but this result was not mirrored in the gastrocnemius. Several mechanisms exists that could explain the difference in muscle carnosine loading between soleus and gastrocnemius, namely, increased insulin sensitivity in the soleus (62) and a preferential insulin-induced translocation of Na^+^/K^+^-ATPase subunits in oxidative fibers (e.g., the soleus) over glycolytic fibers (e.g., the gastrocnemius). Intramuscular Na^+^-K^+^-pump activity can also be stimulated by caffeine (58), meaning that co-supplementation of caffeine and BA may enhance muscle carnosine loading through this mechanism as well. While data surrounding dietary confounders, such as meal co-ingestion or caffeine co-supplementation, on the *TauT*-mediated transport into muscle and how this may impact intramuscular carnosine concentrations is lacking, more research in this area may allow development of supplementation strategies to enhance carnosine uptake into the skeletal muscle.

### Influence of Exercise

Increases in muscle carnosine content have been hypothesized to be an adaptation to long-term high-intensity training as demonstrated by higher values in bodybuilders (63) and trained sprinters (64). It remains unclear whether this is due to genetic predisposition, an adaptative response to the training stimulus, or secondary to differences in muscle fiber type composition. Certainly, a greater number of type II muscle fibers are shown in resistance and sprint-trained individuals while muscle carnosine has a higher content in type II glycolytic fibers compared to type I oxidative fibers (20, 65). These data have also been attributed to a greater increase of dietary BA (via increased meat intake) or to chronic steroid use in these populations [the anabolic effect of androgens may play a role in muscle carnosine metabolism (35, 66)], although the true effect of these factors remains unclear. Nonetheless, chronic training is often cited as a determinant of increased muscle carnosine content (37).

Despite cross-sectional data suggesting an adaptive role of muscle carnosine in response to training, most short-term (4–16 weeks) exercise training protocols have, however, failed to modify intramuscular carnosine content (65–69). Nevertheless, a recent study demonstrated that, independent of BA supplementation, 12-weeks of high-intensity interval training in vegetarians can increase muscle carnosine content in the absence of any dietary BA intake (70). This indicates that an increase in muscle carnosine synthesis occurred despite no ingestion of BA, meaning there may have been an increase in endogenous BA production, although this was not measured. The major part of these increases was attributed to an increase in muscle carnosine metabolism, although it is unclear what the mechanisms are since no changes in the expression of genes involved in carnosine metabolism were shown. This may have been due to the timing of muscle sampling relative to the training sessions; gene expression was determined from muscle biopsies taken at one timepoint 72 to 96 h following training. The possibility that changes in gene expression occurred at different time points following exercise cannot be excluded while replication of these data in omnivorous individuals is also warranted.

Despite clear evidence that exercise can influence muscle carnosine homeostasis (70), no study to date has shown significantly enhanced muscle carnosine loading when BA supplementation was performed in conjunction with a specific training program (45, 65, 69, 71). The reasons for these findings are unclear since none of these studies measured changes in the enzymes, proteins and transporters involved in muscle carnosine regulation. Furthermore, differences in exercise intensity and modality, training duration and dietary habits challenge the ability to isolate why any given individual study showed no combined effect of training and BA supplementation on muscle carnosine increases. Greater increases in muscle carnosine content were shown in trained vs. untrained muscles following 23 days of supplementation at 6.4 g·day^−1^ despite the athletes not being put through a specific training protocol (72). Kayakers showed more pronounced gains in muscle carnosine in the deltoid muscle compared to the soleus and gastrocnemius, whereas the reverse pattern was seen in cyclists. Swimmers, whose exercise task requires both upper and lower-body training, had significantly higher increases in carnosine in both the deltoid and gastrocnemius compared with non-athletes. These results imply a role of training on muscle carnosine metabolism, although a lack of gene or protein measurements hinders mechanistic interpretation of these findings. The authors suggest an increased delivery of BA to the working muscle cells or a possible contraction-induced stimulation of the BA transporters may have contributed to these differences, but data to support or refute this hypothesis is currently unavailable. Overall, little is currently known on how exercise may influence muscle carnosine metabolism. Consequently, more mechanistic studies are required to determine the effects of both an acute exercise bout and chronic training on the major regulators of carnosine content. This will provide information as to whether there is any physiological relevance to ingesting BA at specific timepoints relative to exercise training.

### Co-supplementation of Histidine With Beta-Alanine

Carnosine is formed by BA with L-histidine and is therefore dependent on the availability of both of these amino acids (28). Blancquaert et al. (40) showed a significant depletion of muscle histidine content following 23 days of BA supplementation. The authors speculated that this reduced muscle histidine availability could be the reason for an impaired efficiency of carnosine loading with BA as supplementation is extended over time (16, 40). While co-supplementation of BA and histidine in their study did avoid the depletion of muscle histidine stores, intramuscular carnosine content was unaffected when compared to supplementation with just BA. These results corroborated previous findings of Harris et al. (1) that showed no additive effect of BA and histidine supplementation on muscle carnosine changes. A number of subsequent studies reported no influence of BA supplementation on histidine content, (23, 24, 50), a finding that was since corroborated by meta-analysis of available data (15). Differences in dietary intake may explain some of the differences between these studies since the average American diet generally contains more protein than the typical Belgian diet (23), although this remains highly speculative. Currently, evidence suggests that histidine depletion is not a limiting factor to muscle carnosine synthesis, meaning co-supplementation of BA with histidine (or carnosine supplementation) will not further augment any increases seen in muscle carnosine content. Regardless, future work should continue to explore the effect of one's diet on muscle histidine content and whether prolonged supplementation with BA at high doses leads to histidine depletion in muscle.

## Current Recommendations and Future Investigation

Chronic BA supplementation increases the intracellular content of carnosine in skeletal muscle and can subsequently improve sports and exercise performance. Current recommendations (13, 15, 73) based upon the available evidence suggest ingesting 3.2 to 6.4 g·day^−1^ of BA for 4–24 weeks. To avoid the uncomfortable feeling of paraesthesia, it is recommended to fraction daily doses into 0.8 and 1.6 g doses at intervals of 3 to 4 h. Adhering to this supplementation regimen will minimize side-effects and lead to significant gains in muscle carnosine content that can benefit exercise performance. However, since greater increases in muscle carnosine are associated with greater exercise benefits (16), herein we have discussed several factors which may optimize the gains achieved using these current recommendations although further work is necessary to elucidate the most achievable methods by which to optimize the muscle carnosine response to BA supplementation.

Studies have shown that chronic supplementation with BA may lead to upregulation or downregulation of the genes associated with carnosine metabolism, although results are contrasting. It would be of interest to determine what the acute response (i.e., timecourse) of these transporters, proteins and enzymes are following a standard BA dose, and whether these changes reflect or predict the longer-term changes in muscle carnosine content. In particular, evidence suggests that the BA transporter *TauT* likely exerts an important role in the observed changes during supplementation. Several avenues exist to test the importance of this transporter including co-supplementation with taurine [since this downregulates *TauT*; (74)] or caffeine, and *TauT* knockout animal models. Further investigations should also focus on the independent influence of insulin and exercise on muscle carnosine metabolism to determine the exact mechanism(s) by which diet and physical activity may optimize increases in muscle carnosine content with BA supplementation. Investigation into different formulations of BA is needed to determine if sustained-release tablets can enhance muscle carnosine increases with chronic supplementation. It is also crucial to determine whether small gains in muscle carnosine content above those generally shown, induced by manipulation of some of these modifiable factors, do indeed lead to worthwhile improvements in performance.

## Conclusions

**Figure 2:**
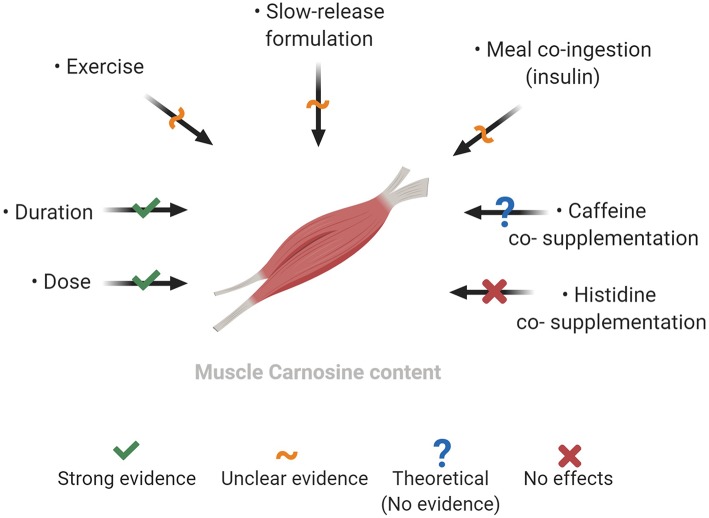
Factors which may modify the increases in muscle carnosine content with beta-alanine supplementation. Created with BioRender.

Several modifiable factors may optimize the muscle carnosine response to BA supplementation, of which the dose and duration are the strongest known moderators (Figure 2). Other factors may optimize increases, particularly during the initial weeks of supplementation, including supplement formulation, ingestion timing in relation to meals and exercise, although stronger evidence to support this is needed. As it stands, more mechanistic work is necessary to elucidate whether BA supplementation can lead to greater muscle carnosine gains above those shown with current recommendations.

## Author Contributions

BS and PP are responsible for the conception of the work. PP, FM, FR, GB, NG, and BS are responsible for the initial writing of the manuscript. FM created the figures. CK and ED reviewed and made significant contributions to the manuscript. All authors approved the final version of the manuscript.

### Conflict of Interest Statement

BS has previously received financial support from Natural Alternatives International (NAI), a company that produces BA, to undertake a study unrelated to this review. CK has previously received a donation of BA and placebo from NAI for a study that was also unrelated to this review. NAI has also provided BA supplements free of charge for further experimental investigations and supported open access page charges for numerous publications involving the authors. NAI have not had any input (financial, intellectual, or otherwise) into this review. The remaining authors declare that the research was conducted in the absence of any commercial or financial relationships that could be construed as a potential conflict of interest.
